# Novel median thoracic expansion for asphyxiating thoracic dystrophy

**DOI:** 10.1093/jscr/rjac345

**Published:** 2022-08-11

**Authors:** Wenlin Wang, Weiguang Long, Yang Liu, Bin Cai

**Affiliations:** Department of Chest Wall Surgery, Guangdong Second Provincial General Hospital, Guangzhou 510317, China; Department of Chest Wall Surgery, Guangdong Second Provincial General Hospital, Guangzhou 510317, China; Department of Chest Wall Surgery, Guangdong Second Provincial General Hospital, Guangzhou 510317, China; Department of Chest Wall Surgery, Guangdong Second Provincial General Hospital, Guangzhou 510317, China

**Keywords:** asphyxiating thoracic dystrophy, median thoracic expansion

## Abstract

There are several surgical techniques for asphyxiating Thoracic Dystrophy (ATD), and various techniques have various indications, but no one has studied this problem in the past. We designed a new procedure for the type of ATD with narrow and cylindrical thorax, and clinical results show that this method is reasonable for this type of patient.

## INTRODUCTION

Asphyxiating Thoracic Dystrophy (ATD) is an extremely rare and dangerous genetic disease [1]. Due to the narrow thorax and severe compression of the lungs, most patients die in the first year after birth [2, 3]. We recently performed an operation on a 4-month-old patient. A special procedure was designed and used to increase the thoracic volume, and good results were obtained.

## CASE REPORT

**Figure 1:**
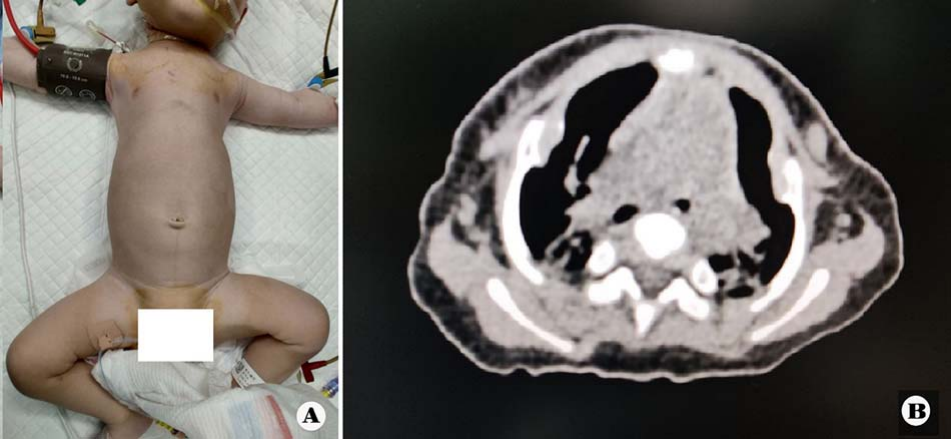
(**A**) Thorax appearance before operation, and (**B**) CT scan image of thorax before operation.

**Figure 2:**
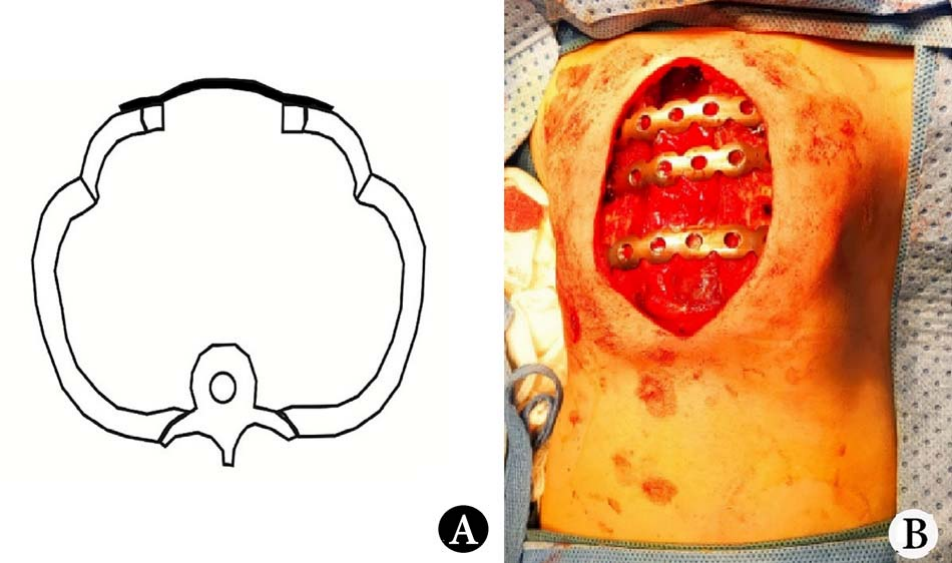
(**A**) Schematic diagram of operation, and (**B**) operative picture.

**Figure 3:**
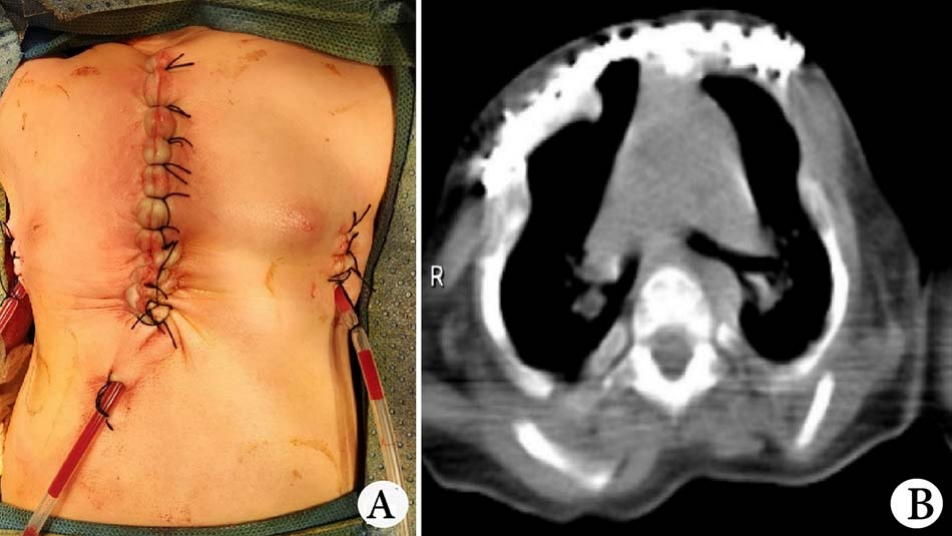
(**A**) Thorax appearance after operation, and (**B**) CT scan image of thorax after operation.

The patient was a female infant born at term with no family history. After birth, the patient developed cyanosis and dyspnea immediately, and emergency intubation and ventilation were performed. One week later, she was weaned and oxygen was given with mask. Oxygen saturation could be maintained basically, but it would decrease when eating and crying. She was eventually diagnosed as ATD and admitted to our hospital to accept surgery four months after birth. Physical examination showed that her chest circumference was 35 cm, and her thorax was narrow and small (Fig. 1A). CT scan revealed that her thorax was deformed, the lateral chest walls were slightly sunken and the lung compression was obvious (Fig. 1B). The operation was performed under general anesthesia. Midsternotomy was made. After the two sternal halves were expanded, three steel bars were placed and fixed on the anterior surface of the sternum (Fig. 2A and B). The bars were covered with pectoralis major muscles, and the skin incision was eventually sutured (Fig. 3A). The chest circumference increased to 40 cm, and the respiratory function was significantly improved postoperatively. Mechanical ventilation was continuously used for 3 days after operation. After weaning, the mask was used for intermittent oxygen supply and stopped 35 days after operation, with the oxygen saturation maintaining above 91%. The patient was discharged 41 days postoperatively. She was followed up for 3 months. There was no hypoxia when calm, but mild hypoxia when crying, which would be relieved after quiet. CT examination 3 months after operation showed that the shape of thorax is improved (Fig. 3B).

## DISCUSSION

Lesions of ATD involve multiple organs, but the main manifestation is narrow chest [3, 4]. The only effective way to treat it is operation. The main purpose of the operation is to increase the thorax volume. There are many methods reported in the literature, which can be summarized into three categories: median thoracic expansion (MTE) [1], lateral thoracic expansion (LTE) [2] and orthopaedic surgery [3, 4]. Different authors have used these methods to treat ATD, but no one has discussed the indications of these methods [1–4].

By April 2022, we had operated on 32 patients with ATD totally. We found that ATD could be divided into two types according to the pathological changes of thorax: type I, the thorax was roughly columnar, and there were slight depressions on the lateral chest wall; Type II, the thorax was bell-shaped, and there were serious depressions on the lateral chest wall. If there is obvious depression on the lateral chest wall, MTE will aggravate the depression; it is not suitable for type II ATD patient. When LTE is used, the chest wall depression will bring a lot of inconvenience to the operation, so it is also not suitable for type II ATD patient. Contrarily, orthopaedic surgery is specially designed for depression, so it is suitable for type II ATD patients. For type I ATD patients, the depression is not obvious, and thus, it is suitable to use MTE and LTE, but not orthopaedic surgery. Our patient was typically type I ATD patient, and we choose the MTE for her, but our method is different from the reported methods [1].

The reported MTE is to put materials between the two sternal halves, which increase the thorax volume by increasing the circumference [1]. We found that if the space between the sternal halves were not occupied by materials, it would help to increase the thorax volume. Therefore, we put the steel bars on the anterior surface of the sternal halves, leaving the gap in the middle empty (Fig. 2A). This procedure can not only increase the chest circumference but also increase the space between sternal halves. We think that it will help to increase the chest volume.

Our procedure has significantly improved the patient’s respiratory function, which shows that this method is effective. We believe that it is a reasonable choice for type I ATD patient.
